# A common future amidst unknowns: an *NSR* forum on polar science

**DOI:** 10.1093/nsr/nwae379

**Published:** 2024-10-24

**Authors:** He Zhu

## Abstract

The final frontiers on our planet challenge humans’ survival as much as our ambition. The Arctic and Antarctic areas together exert enormous influence on the climate of the whole planet, as they contain 87% of its fresh water, 90% of its ice and snow, 90% of its permafrost and 69% of its glaciers. However, our lack of understanding of changes in the polar regions, such as melting ice sheets, results in major uncertainties in our estimates and predictions with regard to rising sea levels and other effects of climate change. For example, as the Arctic region is warming up two to four times faster than the global average, Eurasia now experiences colder winters. As its mechanism is yet to be elucidated, forecasts of extreme weather events in China, which aim to avert severe damages, remain inadequate. In order to raise awareness of polar science, *National Science Review* invited Dr. Dake Chen of the Second Institute of Oceanography, Ministry of Natural Resources (MNR) to organize a forum discussion with five Chinese experts to explore these crucial topics.

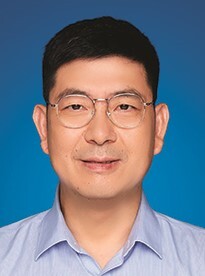

**Jianfang Chen** (陈建芳)

Professor, Second Institute of Oceanography, MNR

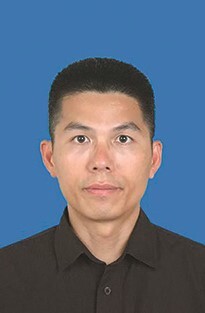

**Ruibo Lei** (雷瑞波)

Professor, Polar Research Institute of China, MNR

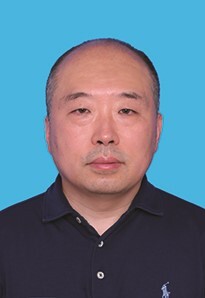

**Jiping Liu** (刘骥平)

Professor, School of Atmospheric Science, Sun Yat-sen University

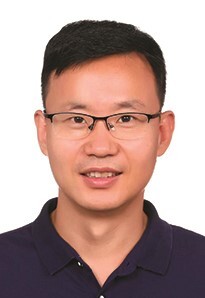

**Qinghua Yang** (杨清华)

Professor, Sun Yat-sen University and Southern Marine Science and Engineering Guangdong Laboratory (Zhuhai)

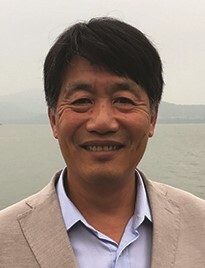

**Meng Zhou** (周朦)

Professor, School of Oceanography, Shanghai Jiao Tong University

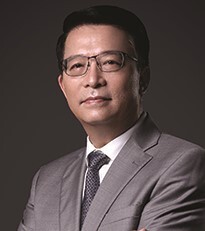

**Dake Chen** (陈大可) (Chair)

Professor, The Second Institute of Oceanography, MNR and Southern Marine Science and Engineering Guangdong Laboratory (Zhuhai)

The changes in the Arctic are causing extreme winter events in mid-to-low latitude regions such as China. The complex connections between the Arctic system and the rest of the world are strengthening.

—Ruibo Lei

**Chen DK:** We all have dedicated many years of research to polar regions and today we'd like to gather some insights on the related topics. First, let's discuss the issue of the rapid warming of the Arctic. Prof. Lei, would you like to give an overview?

**Lei RB:** The Arctic now experiences amplified warming and there is still some controversy regarding its driving mechanism. First, is it a local effect or caused by the energy transport from lower latitudes? A local effect could be attributed to a positive feedback loop that consists of more ice-melt during the summertime and less reflected sunshine, more absorbed heat and even more melting. In addition, clouds may play an interesting role, as more evaporation results in more clouds and larger downward longwave radiation. Heat transfer into the Arctic occurs either through atmospheric or through oceanic circulations. Atmospheric transfer is not believed to accumulate significantly because it is a process of short-term impact. However, the warm water from the Atlantic Ocean may increase water and air temperature, and reduce the amount of sea ice, especially in the Atlantic section.

Another crucial issue regarding the Arctic Ocean is that it is surrounded by land, so the effects of melting permafrost contribute to the increase in greenhouse gases in the Arctic by up to 30%. On the other hand, the changes in the Arctic are causing extreme winter events in mid-to-low latitude regions such as China. The complex connections between the Arctic system and the rest of the world are strengthening.

**Figure 1:**
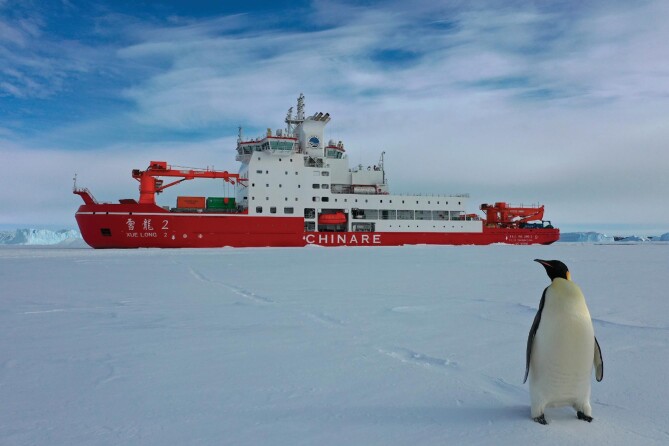
The icebreaker research vessel, Xuelong 2, built in China with PC3 icebreaking capabilities, was launched and applied to scientific investigations in the Southern Ocean in 2019, significantly enhancing China's investigation capabilities in polar waters covered by ice. (Courtesy of Rong Huang)

**Chen DK:** We probably should talk a bit more about the amplified warming effects in the Arctic. Prof. Liu, what is your view on this? Is there a positive feedback loop behind the amplification?

**Liu JP:** The explanation for the amplified effect used to be the increase of heat transfer to the Arctic as a part of climate change. Recently we performed numerical simulations with a climate model and compared scenarios of fixed versus evolving sea ice amounts. Our results show that the local changes in sea ice amounts and the sun reflection associated with it play a dominate role in the Arctic amplification. The heat transfer from low latitudes does exert some impact but only to a minor extent. That is where our research stands currently, and new ideas may develop as our understanding of the Arctic system improves in the future.

**Chen DK:** Next we discuss the ice sheets in Greenland and Antarctica and how they affect the global sea level. Prof. Liu, would you like to start?

Incrementally, every 1 cm rise [in sea level] would endanger the lives of ∼6 million people worldwide through increased severe events in coastal regions.

—Jiping Liu

**Liu JP:** Antarctic and Greenland ice sheets together hold 68% of the fresh water on Earth. The mass of the ice sheets can be changed by factors such as snow accumulation, evaporation, water streams, and the melting and collapsing of ice shelves or ice tongues. We currently tackle this complex problem through measurements of satellite or aerial remote sensing. In the extreme case of entire ice sheet melting, Greenland and Antarctica would contribute 7.4 and 58 meters to global sea level rise, respectively. Incrementally, every 1 cm rise would endanger the lives of ∼6 million people worldwide through increased severe events in coastal regions. Past measurements tell us that the Greenland sheet was mostly stable until the 1970s and then it started to lose mass. The decrease of the Greenland sheet has accelerated in the past three decades and has contributed 12 millimeters to the rise in sea level. The western Antarctic sheet experienced a similar change, and has contributed ∼7 millimeters to sea level rise. In recent years, the mass of the Antarctic sheet has increased largely due to increased heavy snowfall in eastern Antarctica. This unexpected change highlights the lack of understanding and uncertainty with regard to changes in the ice sheets. Since the melting ice sheets are the driving force of rising sea levels, the Intergovernmental Panel on Climate Change (IPCC) has placed great emphasis on the mass balance and dynamics of the ice sheets. In particular, the eastern part of Antarctica stands out as a major unknown due to the lack of observations, which hinders the development of climate models. Furthermore, it has been a challenge for the dynamical model of the ice sheet to represent its rapid changes in recent years. In the past, we used to consider its changes to be noticeable on the order of hundreds to thousands of years. Now we are witnessing changes within decades. New ideas are required when we construct models to reflect ice sheet interactions with the atmosphere, ocean and sea ice. Only through accurate models of the ice sheets can we provide accurate predictions of the rise in sea level. Our current Earth system models predict that, at the end of the 21st century, the Greenland ice sheet will lead to a 0.15 m rise and the Antarctic ice sheet will lead to a 0.12 m rise. The last issue related to the melting ice sheets is that it may weaken global ocean overturning circulation, affecting the marine ecosystem and, therefore, the amount of CO_2_ uptake by the ocean.

**Figure 2:**
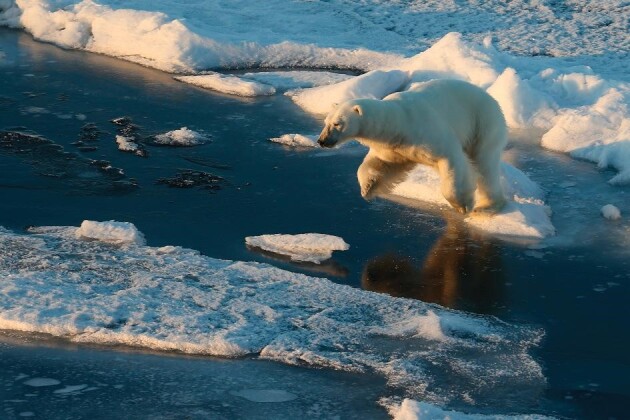
A polar bear migrates over the summer ice floes in the Arctic Ocean. (Courtesy of Rong Huang)

**Chen DK:** Ice sheets in polar regions have received considerable attention globally and the core question revolves around the existence of a so-called climate ‘tipping point’. Will we reach such ‘a point of no return’? How much uncertainty is there in our current projection of ice-sheet and sea-level changes? Hopefully we will be able to answer these questions through more scientific polar expeditions, more systematic observations and more realistic models. Now, let's move on to the next topic of polar ecology. As we all know, both polar regions consist of unique ecosystems. The hidden habitats under polar ice sheets may conceal extraordinary ecological and biological secrets. Prof. Zhou, would you like to start on this topic?

**Zhou M:** Both polar regions possess rich biological resources supported by highly productive ecosystems. But the period of high productivity only lasts 2 to 4 months during the summer, while for ∼8 months of the year, photosynthetic energy and productivity are extremely low. Naturally we need to ask how polar biological communities use the energy obtained in four summer months to sustain their survival for a year. For the terrestrial ecosystem at high latitudes, animals spend winter months in hibernation or with stored foods. In the ecosystem of the Southern Ocean, krill occupy a central role as they feed on algae in spring and summer and they are fed on by higher trophic-level organisms, such as fish, penguins, seals and whales. Unlike terrestrial animals, krill do not hibernate in fall-to-winter months, and other polar marine animals in the food chain above krill are, therefore, also active in fall-to-winter. We estimated the annual survival rate of krill to be less than 1 in 100. Thus, if the approximate age for a krill fishery is 4 years old, the 4-year survival rate of krill is less than 1 in 100 million. The bio-energy source of this enormous amount to feed krill biomass is yet to be determined.

If there are subglacial lakes sealed underneath the ice sheet, there might be living organisms that have been isolated from our world for 2 million years.

—Meng Zhou

There are significant differences in marine dominant species, and their behaviors, between the two polar regions. One of the keystone species in the Arctic marine ecosystem is *Calanus finmarchicus*, a copepod species with a maximum length of 2–4 mm. In the spring, massive amounts of *C. finmarchicus* feed on algae and synthesize lipid that turns the shelf seas to dark red. One of the keystone species in the Antarctic marine ecosystem is *Euphausia superba*, also known as Antarctic krill, with a maximum length of 7–8 cm. The Antarctic krill can form a super aggregation of 10–20 km with an abundance of 10^4–5^ # m^−2^. Both are critical prey for fish larvae and higher trophic-level animals. In the north, *C. finmarchicus* appear in the spring and grow through the summer. Then they descend to 600–800 m in later summer and enter hibernation to avoid other animals during fall and winter. In the south, Antarctic krill continue actively feeding through the austral winter. Nevertheless, for both *C. finmarchicus* and krill, food sources and webs of winter ecosystems remain poorly understood. In the past, scientists hypothesized that both dissolved and particular organic matters in the water column and sediments produced from summer months provide food for winter ecosystems through microbes, but these hypotheses have never been proven by balancing biomass fluxes between functional groups or trophic levels. This lack of understanding of the ecosystem results from the lack of observations in the months without light, as our research vessels cannot stay through those months due to thick solid sea ice and lack of sampling methods. Since we do not know how these marine animals survive in the period of fall and winter, we do not know the initial conditions of their number and biomass and their spatial distributions in the early spring. This is the primary challenge we face in the research of polar ecology, and ultimately the modeling of ecosystems and climate change. So far, Chinese scientific expeditions have focused on the period between July and September in the Arctic Ocean and between December and March in the Southern Ocean. Moving forward, I'd like to see our oceanographic surveys and studies in these remote waters applied to the winter periods between December and March in the Artic Ocean and between April and November in the Southern Ocean.

In Antarctica, another great opportunity for biological research awaits us within the ice sheet itself, which is approximately 1.8 km thick and up to 2 million years old. There may be microorganisms frozen in this ice. If there are subglacial lakes sealed underneath the ice sheet, there might be living organisms that have been isolated from our world for 2 million years. If we can indeed collect samples and gain knowledge of the evolution of life over such a long period of time, it'd greatly help humanity understand the origin and evolution of life on Earth and even in the universe in general. One particularly exciting activity in China that is gaining attention around the world has been our effort to drill for ice cores from Dome A on the Antarctic ice sheet. If we can acquire intact samples of ice cores from the bottom of the ice sheet, air bubbles or microorganisms sealed in the ice cores may reveal the dates and compositions of the atmosphere and microbes from that period and tell us about the evolution of the environment and life forms on Earth. In addition, possible volcanic activities under the ice sheet could sustain warm water and chemosynthetic energy. Some of our colleagues speculate that exotic life forms could have evolved in such an isolated and extreme environment.

**Chen DK:** You have mentioned two interesting topics and I have two comments. First, the winter months in polar regions may not be completely dark and energy lacking. It surely is a long period of low productivity compared to the ‘big bloom’ of life in the spring, but life must be able to carry over through some sort of strategy and there should be a distinct seasonal cycle. Second, drilling to reach Antarctic subglacial lakes, and taking samples there, has great scientific potential and is now receiving strong support from various funding agencies. Russian researchers started this area of investigation but the samples they retrieved were polluted and thus had limited use. We are trying to develop technologies for clean drilling. If successful, the discovery of microorganisms there alone will constitute a major scientific breakthrough of this century.

**Zhou M:** I agree. Discovery of life forms in or under the ice sheet would be comparable to the discovery of quantum mechanics.

A small fluctuation such as a decrease in CO_2_ absorption in the Southern Ocean may cancel out the annual emissions reduction of China.

—Jianfang Chen

**Chen JF:** I have some comments regarding how marine animals in the Southern Ocean survive the period of low energy and low productivity. First, would it be possible for ocean circulation to carry nutrients and organic matter to the Southern Ocean to support life? Second, can cohorts of some animals, such as krill, survive collectively by feeding on the ones that perish during the winter period?

**Zhou M:** These are great suggestions for further research. In particular, we did look into the possibility of krill feeding on themselves. We once estimated a cumulation of 10 000 krill individuals in one cubic meter of water without any food source. So why would they migrate and accumulate in an apparently self-destructive fashion? Our hypothesis is that strong and healthy ones would consume the weaker ones as they die so that the group may live on till next spring to feed on diatom blooms again. We designed experiments to prove this hypothesis using chemical analysis of the stomach contents of surviving krill, but it was not successful.

**Chen DK:** Now let's move on to our next topic regarding carbon sinks in polar regions. Carbon peaking and carbon neutrality are hot issues on both national and international stages, and we know polar regions play a crucial role in the carbon cycle of the whole planet. For example, the current estimate is that ∼40% of the oceanic uptake of anthropogenic CO_2_ takes place in the Southern Ocean. Prof. Chen, would you like to talk about this?

**Chen JF:** In recent years, leading funding agencies of the state, such as the Ministry of Science and Technology (MOST) and the National Natural Science Foundation of China (NSFC), have placed great emphasis on the national goals of carbon peaking and carbon neutrality. However, current efforts are mostly on coastal carbon sinks, while the open oceans, including the Southern Ocean and the Arctic Ocean, absorb the majority of the carbon going into the ocean (∼1.85 ± 0.95 billion tons). That is orders of magnitude higher than the contribution from the coastal regions. The two polar oceans together make up for more than half of the amount absorbed by the oceans over the world, which is comparable to the CO_2_ absorption of all grasslands and forests in China. The actual absorption occurs partly through physical pumps caused by the water mass moving up and down. With regard to biological pumps, we often use the analogy of an ‘invisible forest in the ocean’ to describe the carbon sink process whereby CO_2_ is absorbed through photosynthesis and sinks into deep water. The challenge we face in understanding this process lies in its uncertainties: a small fluctuation such as a decrease in CO_2_ absorption in the Southern Ocean may cancel out the annual emissions reduction of China. The Arctic plays a smaller role compared to the Southern Ocean, but it is more complex and changes more rapidly because of the rapid sea-ice retreat and the terrestrial influence. Melting permafrost releases CO_2_ while greening of land areas absorbs more CO_2_, both of which are related to Arctic warming amplification. In addition, the Arctic Ocean itself has seen extraordinary changes in terms of ocean currents, water temperature and ecological processes. The large amplitude and great variability of these changes make it particularly difficult to incorporate these changes into climatic and biogeochemical models.

**Figure 3:**
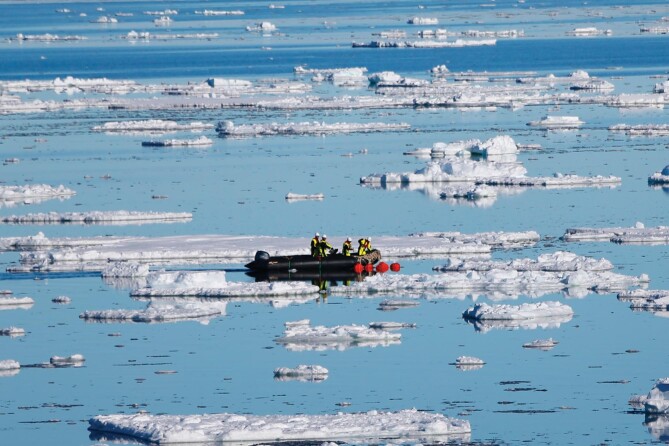
Recovering a mooring observation system in the ice regions of the Southern Ocean is extremely challenging. Chinese scientists are using rubber boats to recover the mooring system in order to obtain year-round oceanographic observation data beneath the ice. (Courtesy of Yongming Sun)

Carbon storage is deeply interconnected with biogeochemical processes. The oceanic path of carbon from sinking to storage can be viewed in the ecosystem as the generation of carbohydrates from photosynthesis and its consumption in the food chain. Ice algae, for example, accumulate under sea ice during autumn to winter months and sink quickly as ice melts in spring and summer. As they sink and reach the intermediate depths of the ocean, they contribute to biological communities consisting of zooplankton, crabs and shrimps. Now, with climate change and rapid reduction of sea ice, carbohydrates from photosynthesis are formed more in shallow waters and feed zooplanktons that support fish and mammals such as seals. Active biological pump and carbon cycling are also observed in topography-induced marine ecosystems such as those near seamounts. One challenge is to understand how they function globally in the framework of climate change.

**Zhou M:** I'd like to mention one topic of carbon sequestration called refractory dissolved organic carbon (RDOC). Transporting DOC to the deep ocean relies on deep convection circulation of ocean water. But the vertical currents in low- and mid-latitude regions only reach a depth of tens of meters. Only in the less-stratified Southern Ocean can severe storms create convection up to 1000 m deep. Such oceanic events occur mainly in the fall and winter, and thus they were not captured by our past scientific expeditions, which usually ended before the fall. Another phenomenon we happened to notice was large amounts of organic sediments on the sea floor. These organic materials can be consumed by other organisms and enter the carbon cycle. This phenomenon is of great significance but was never studied. I hope to see more research on this topic. In addition, various types of algae are transported by ocean currents. Diatom, if not consumed, may reach the sea floor but other types of algae decompose and dissolve in the water column. As ocean circulation and vertical convenction change with the warming climate, settling of particular organic carbon and vertical convection of DOC in the polar regions will also impact the carbon cycle.

**Figure 4:**
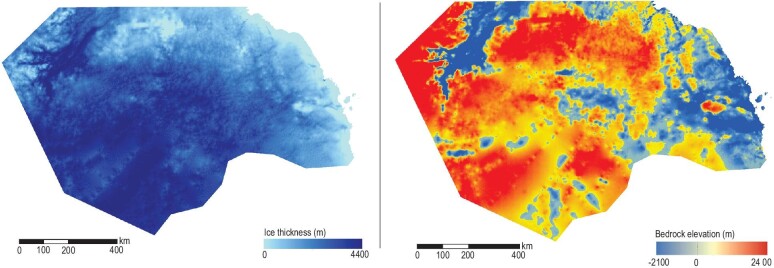
Ice thickness (left) and bedrock elevation (right) in Princess Elizabeth Land (∼0.9 million km^2^), filling the previous largest data gap in Antarctica through airborne ice-penetrating radar survey by China. (Courtesy of Xiangbin Cui)

I am a strong advocate for further enhancing our air transportation and observation capabilities in Antarctica. The completion of Qinling station surely marks a significant step forward and we need to take advantage of it.

—Dake Chen

**Chen DK:** Polar research is of great strategic importance but is also a difficult task because the remote polar regions are hard to reach, and the environmental conditions are extremely tough there. One central component of polar science is the development of research capacity. Prof. Lei, would you like to introduce China's efforts in this aspect?

**Lei RB:** Over the past 40 years, China has gradually built up its capacity for polar expeditions and research, including two large icebreakers, seven research stations and observatories in Antarctica and the Arctic, and a fairly comprehensive suite of polar research equipment. A major upgrade took place in 2019 with the completion of Xuelong 2, an advanced icebreaking research vessel, which strengthened our ability to navigate and study the polar oceans. Now this icebreaker ranks among the top five polar research vessels in terms of annual service time. However, the amount of time spent on oceanographic research by our two icebreakers is still rather limited because of their missions to provide logistic services. Moreover, our research activities over the Antarctic ice sheet are trailing countries such as the USA and Russia due to the lack of aerial capacity. We also need to improve our ability to collect ice and snow samples across extensive areas in a continuous manner. Recently, we reached Dome A over the Antarctic ice sheet with a fixed-wing airplane. This capability will reduce the transit time to and from the Antarctic inland plateau and greatly increase the efficiency of sample collection.

The year of 2024 has seen another major milestone in the building of China's capacity for polar research. On 7 February, Qinling station in Antarctica began operations after 4 years of construction. This beautiful station is located on the Inexpressible Island in the Ross Sea, one of the regions with the worst weather conditions in Antarctica. Some experts from other countries thought we were out of our minds in choosing this location because of the extreme winds, as the location restricts outdoor activities in winter. But on the other hand, how can you study the unique processes of Antarctica without going to such unique locations? Specifically, this location allows us to study the Antarctic katabatic winds that generate and maintain the coastal polynya, and to develop a sustained observing network to investigate and monitor the formation and pathway of Antarctic Bottom water, the largest water mass in the world. In addition, the existence of the polynya and the proximity of the station to the ocean allows for convenient loading.

**Chen DK:** Good points. I particularly want to emphasize aerial capacity. I am a strong advocate for further enhancing our air transportation and observation capabilities in Antarctica. The completion of Qinling station surely marks a significant step forward and we need to take advantage of it. The next topic is sea ice and predictions in this area. Prof. Yang, would you like to give us an overview?

State-of-the-art projections suggest that the Arctic could be ice-free in summertime by the middle of this century, although recent research indicates this could happen even sooner.

—Qinghua Yang

**Yang QH:** Sea ice in the Arctic and Antarctica is experiencing rapid and contrasting changes. Arctic sea ice has been declining for decades, whereas Antarctic sea ice increased slightly until 2014 before entering a phase of rapid decline. A short-lived rebound occurred after 2017, followed by renewed decreases. Notably, satellite measurements recorded Antarctica sea ice at its lowest in 2022 and 2023. Global climate models, which couple atmosphere, ocean, land and sea ice components, are commonly used to predict sea ice variations. However, current modeling frameworks show considerable limitations. For example, they underestimate the rate of the Arctic sea ice decline and struggled to simulate the unexpected increase in Antarctica sea ice before 2014. If climate models cannot accurately reproduce historical sea ice conditions, their reliability in projecting future climate scenarios becomes problematic. A key example is the uncertainty surrounding the prediction of when the Arctic will experience ice-free summers as the climate warms, with a large spread among different models.

**Figure 5:**
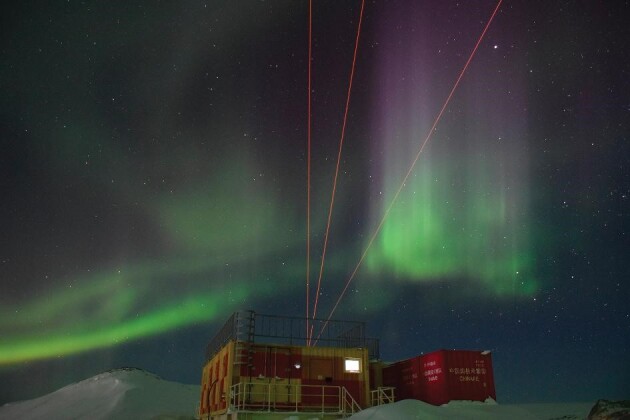
China has deployed four sets of atmospheric detection lidar observation systems at Zhongshan Station, East Antarctica, achieving synchronous ground-based observation of the atmosphere at quasi full elevation (0–110 km) in Antarctica. (Courtesy of Jianjun Liu)

As modeling evolves towards higher spatial resolutions, new challenges arise. Sea ice can no longer be treated as a continuous medium on horizontal scales; instead, it should be modeled as discrete ice floes. This shift necessitates improvements in traditional sea ice dynamical modeling. Recent advances using multi-resolution techniques to conserve computational resources have reached a 1-kilometer resolution for sea ice simulations across the Arctic Ocean. However, estimating the thickness of sea ice remains far more uncertain than estimating sea ice extent, and even models with the best predictive skills continue to show large errors in thickness forecasts.

In terms of future change, state-of-the-art projections suggest that the Arctic could be ice-free in summertime by the middle of this century, although recent research indicates this could happen even sooner. The ongoing decline in Arctic sea ice has significant implications for navigation through the Arctic sea routes. Even if we successfully control carbon emissions with green energy, the accessibility of Arctic passages will be substantially enhanced. This aspect is of strategic importance to the state as it affects global commerce.

**Chen DK:** In the last round of comments, I'd like to invite everyone to briefly describe their expectations of their fields or the bottleneck problems that need to be addressed. Prof. Zhou, let's start with you.

**Zhou M:** Research in polar science, as a frontier in natural sciences, will reveal the mechanism of climate change and the mystery of life on Earth. The natural resources in the polar regions are also crucial to the sustainable development of our world. These aspects, combined with possible navigation through the Arctic, emphasize the importance of polar science. So, I hope scientiests in China will contribute more world-class research in this field.

**Liu JP:** I hope more young scientists in China will focus on physical processes in the polar regions involving atmosphere, ocean, sea ice and their interactions. Efforts in these research areas will allow us to make best use of our growing observational data from the polar regions, improve dynamical models and provide accurate predictions for future developments.

**Lei RB:** I'd like to raise three points. First, polar science is regarded as a regional science, not belonging to one discipline. The growth of this field needs an interdisciplinary approach to integrate physics, biology, ecology and other fields. Second, we need stronger collaboration between observational technology and cutting-edge research in Earth science. Third, I hope more newcomers in polar science at the graduate level will be able to participate in on-site activities.

**Yang QH:** As a scholar in atmospheric research, I'd like to emphasize the growing role of artificial intelligence (AI) in the field of atmospheric science and ocean science. For example, the great impact of AI on weather forecasting is now undeniable. So, I hope in polar science, we will be able to see more observational and modeling work revolutionized by AI.

**Chen JF:** First, as we have developed considerable research capacity for polar science in terms of ‘hardware’, such as vessels and stations, now we need to design and lead scientific programs to demonstrate our contribution to this field. Second, we need to develop more portable technologies tailored for on-site polar observation and research. Third, international collaboration is crucial in this field, and we need to strengthen our partnership with other countries.

**Chen DK:** Today we have conducted an interesting discussion on polar science using the platform provided by *NSR*. The topics we covered may gather controversy as much as interest. I hope this forum will amplify our voice to the broad readership of *NSR* so we may raise public awareness of the roles of polar regions in our world. We'd also like to draw more attention from funding agencies to increase investments. Research projects in this field require large amounts of resources and may also produce great rewards. I still recall the strategic decisions made by national leaders in 1980s to start polar expeditions. In that era of frugal budgets, those projects were carried out in hardship. Now as our resources have grown by orders of magnitude, we ought to expand our investments in this important area. This concludes our discussion and I thank everyone for your participation.

